# How COVID‐19 pandemic may hamper sustainable economic development

**DOI:** 10.1002/pa.2675

**Published:** 2021-03-25

**Authors:** Maruf Yakubu Ahmed, Samuel Asumadu Sarkodie

**Affiliations:** ^1^ Business School (HHN) Nord University Bodø Nordland Norway

## 1 COVID‐19 ECONOMICS

**FIGURE 1:**
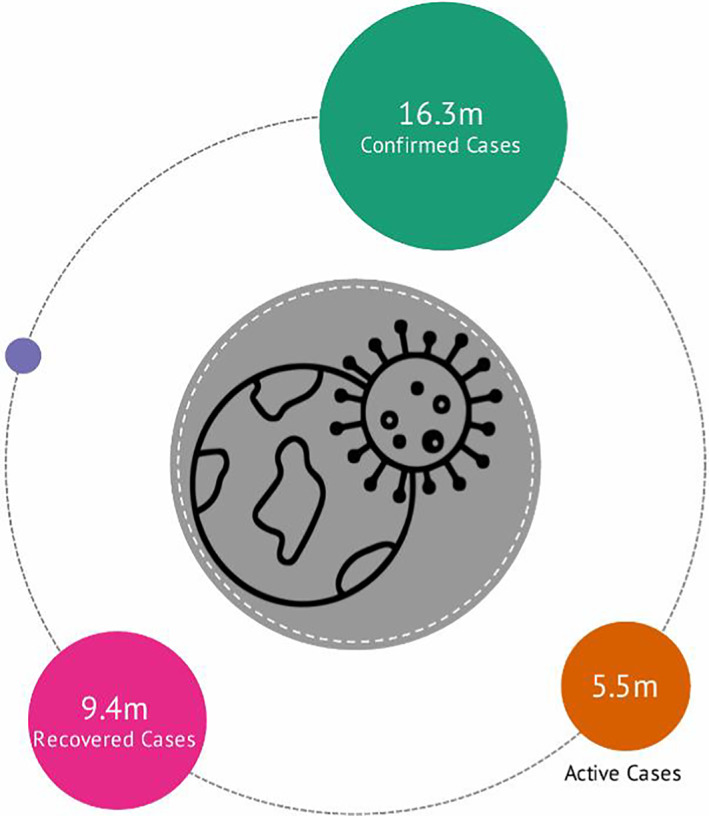
Global reported cases of COVID‐19 pandemic. Data source: Lauren (2020); Knoema (2020)

The novel coronavirus disease (COVID‐19) was first reported as a cluster of pneumonia in the city of Wuhan in the Hubei province of China in December 2019. This was eventually identified to be severe acute respiratory syndrome (SARS‐Cov‐2) infectious disease with clinical symptoms of dry cough, fever, tiredness, pneumonia, and respiratory disorder that can lead to death in severe cases (Sarkodie & Owusu, 2020a). The World Health Organization (WHO) declared COVID‐19 as public health emergency of international concern on 31 January 2020, after spreading from China to 24 countries. The declaration was intended to strengthen the coordination of appropriate public health responses to prevent the rapid spread of the disease. However, the significant travel connections around the world aided more infected individuals to arrive in international locations before appropriate control measures were put in place (Magazzino et al., 2021). The earlier reported number of infected persons as of 20 February 2020 stood at 76,496 cases including China (75,245), Diamond Princess cruise ship (634), South Korea (104), and other countries (513) infections (Sarkodie & Owusu, 2020b). WHO declared the outbreak as a pandemic on 11 March 2020, due to the alarming rate of spread and global severity (CDC, 2020). As of 27 July 2020 (see Figure 1), the COVID‐19 outbreak had spread globally infecting estimated 16,305,273 persons—with 9,397,505 recoveries—5,509,025 active cases and 654,777 reported deaths in approximately 188 countries (Lauren, 2020).

The COVID‐19 virus devise means of transmission through direct and indirect, or close contact with infected secretions including saliva or respiratory droplet expelled from cough or sneeze. The disseminated infectious droplet nuclei and infectious droplet expelled can contaminate the surfaces and objects creating fomite (WHO, 2020). The lack of effective vaccine has forced central government across the globe to implement a series of lockdown intervention including social‐distancing measures, shutdown of travel from in and out of the country. These large‐scale lockdowns and restrictions by countries have disrupted the whole economic structure—leading to a drastic fall in foreign direct investment (FDI), gross domestic product (GDP), and export of goods and services (EGS) globally (Knoema, 2020). The COVID 19 pandemic outlook is highly uncertain, with perspective dependent on the duration of the health crisis. Global FDI was estimated to fall by 4000 basis points (bps) in the 2020 fiscal year, resulting in a low global FDI value of $1 trillion compared to $1.54 trillion in the 2019 fiscal year. FDI is expected to further fall by 50–100 bps in the 2021 fiscal year, representing 600 bps decline since 2005, from $2 trillion to less than $900 billion (United Nation Conference on Trade and Development [UNCTD], 2020).

**FIGURE 2:**
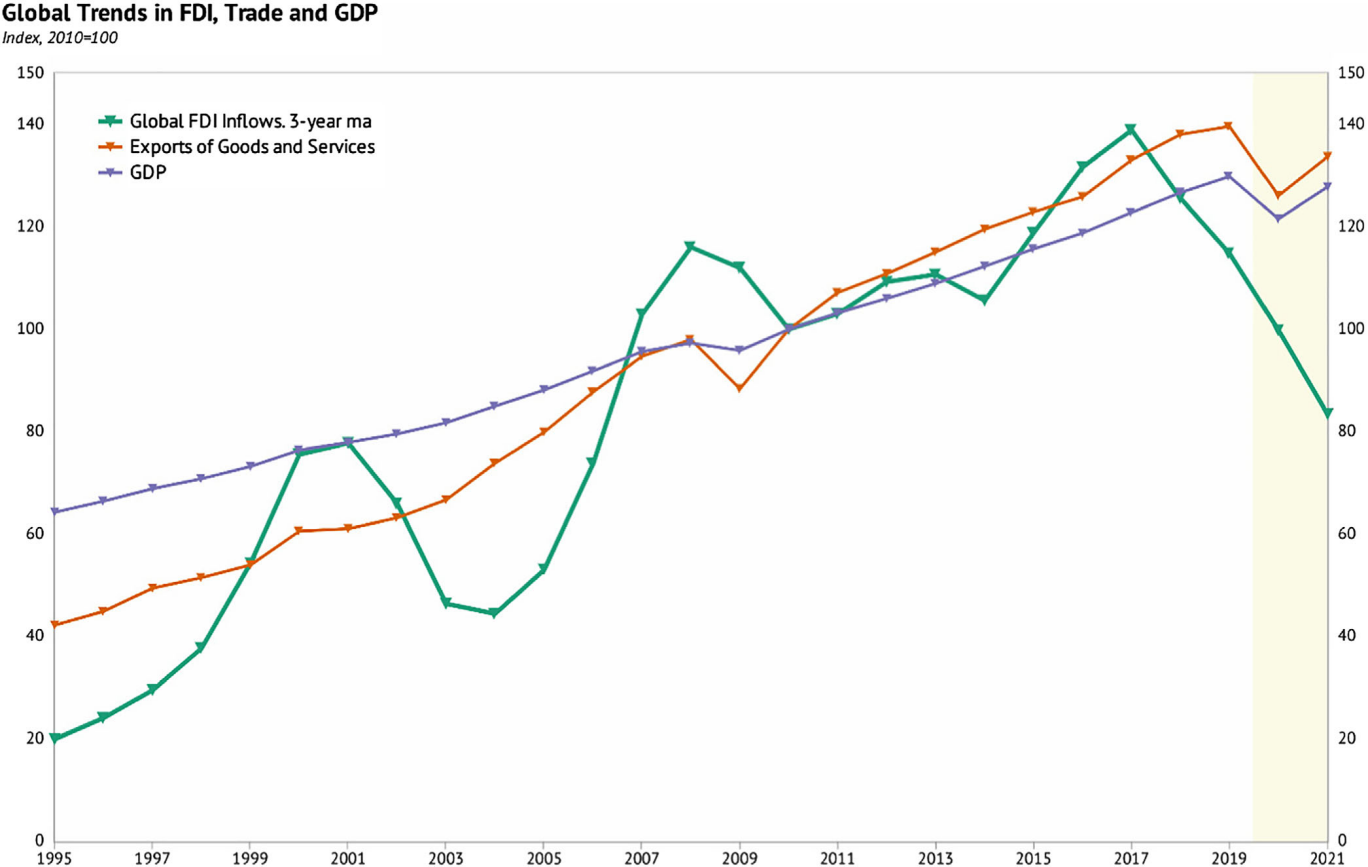
Effect of COVID‐19 pandemic on global FDI, trade, and GDP. Data source: UNCTD; Knoema (2020)

Figure 2 shows upward and downward movements of global FDI inflow over 14 years, with downward trend in 2019 and first half of 2020 far below both GDP and trade, this downward trend is expected to continue in 2021. These global economies are expected to register a weak performance more than the global financial crisis of 2008–2009. The demand and supply, trade, and finance in both developed and developing markets were set to fall sharply in the year 2020. Global GDP is estimated to reach its deepest global recession in decades, declining by 520 bps 2020, despite large‐scale macroeconomic policies to soften the economic crisis—which far exceeds the policies enacted during the 2008–2009 financial crisis (World Bank, 2020). The advanced economies are expected to shrink by 700 bps, with China's market forecast to slow growth to 10 bps—the lowest in four decades—and emerging market and developing countries (EMDC) were estimated to fall by 250 bps in 2020. EMDC will be hard hit by the sharp decline in China's growth and decline in international commodity price resulting from falling in global demand on the international market, particularly crude oil (World Bank, 2020). The adverse spillover resulted in more than 90% fall in per capita income in EMDC economies in 2020. Figure 2 shows a consistent upward trend in GDP from previous years with a sharp fall in December 2019, with a stable rise in 2020 to mid‐year. Most central banks in developed countries have cut down policy rate and taken appropriate measures to ensure adequate liquidity and maintain confidence of capital owners. For example, the US is reported to have instituted 100% policy cut among 162 nations due to the COVID‐19 pandemic (Sarkodie & Owusu, 2020c).

**FIGURE 3:**
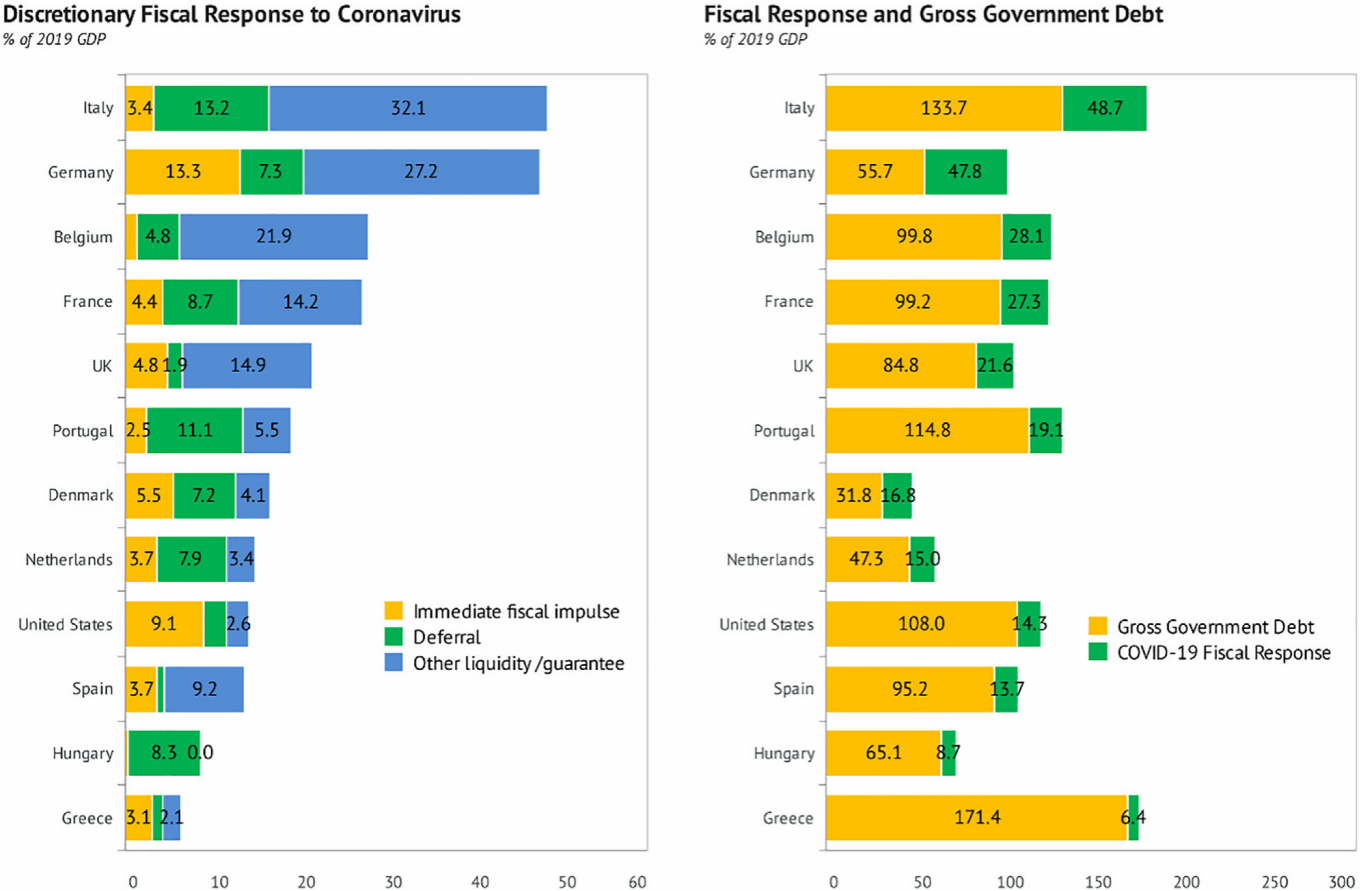
Fiscal response in largest EU economies and the United States on COVID‐19 pandemic. Data source: Bruegel; Knoema (2020)

Figure 3 shows the response of governments around the globe to the pandemic by allocating fiscal policy measures. Among the largest economies in the European Union and the United States, the 48.7% fiscal response in the form of immediate fiscal impulse, deferral loan, and other liquidity/guarantees from the Italian government stood tall among these countries, followed closely by Germany (47.8%) (see Figure 3). As of April 2020, large fiscal support through revenue and expenditure of 350 bps of GDP of Great twenty (G‐20) Economies were provided in response to COVID‐19. The public sector liquidity support such as loans and guarantees were introduced to support financial and non‐financial companies and small‐medium scale enterprises totalling $4.5 trillion globally. Loan and guarantee support in European countries stood at 16.7% of EU GDP. The average public debt across the globe has plateaued because of the crisis, global public debt level—at 8300 bps of global GDP in 2019 and was estimated to increase by 1300 bps to reach 9460 bps of global GDP in 2020. As depicted in Figure 3, if the COVID‐19 pandemic lingers, the top five countries with highest gross government debt will include Greece (171.4% of 2019 GDP), Italy (133.7% of 2019 GDP), Portugal (114.8% of 2019 GDP), the United States (108% of 2019 GDP), and Belgium (99.8% of 2019 GDP). High debt and rising cost of capital will make it difficult for governments to implement countercyclical fiscal economic policies (International Monetary Fund, 2020).

**FIGURE 4:**
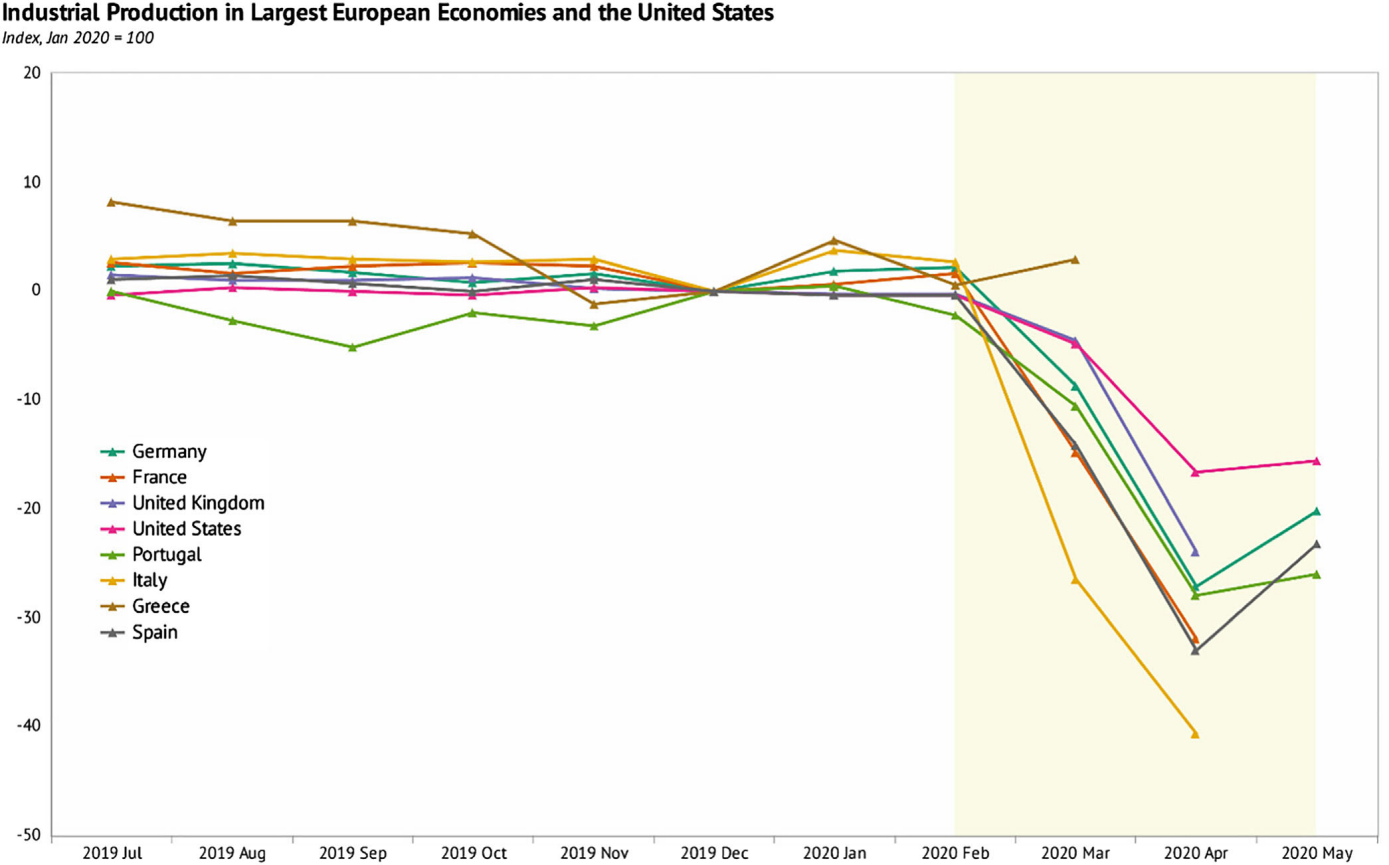
Industrial production affected by COVID‐19 pandemic in largest EU economies and the United States. Data source: World Bank global economic monitor; Knoema (2020)

Besides, the COVID‐19 pandemic has caused global trade to fall more in 2020 than it did in the 2008–2009 financial crisis. International trade involving goods and services is volatile and decline sharply in time of crisis. The global trade market is estimated to fall by 1340 bps in the first half of 2020 and travel restriction due to COVID‐19 led to a fall in the tourism sector accounting for about 650 bps in global food and service supply (Word Bank, 2020). The collapse of international air traffic and stringent border control resulted in a sharp increase in air cargo cost, affecting the major industries that provide just‐in‐time service delivery of already‐made foreign products and delay in supply of automotive and electronics critical to industrial input (Baldwin & Tomiura, 2020; Haren & Simchi‐Levi, 2020). The global pandemic severely affected industrial production—leading to economic shock with future consequences. As observed in Figure 4, COVID‐19 restrictions gravely affected the United States and leading EU economies namely Italy, Spain, France, Portugal, Germany, United Kingdom, and Greece. Figure 2 shows the annual upward trend of goods and services with a sharp fall at the end of 2020 when the outbreak of COVID‐19 started—and slow growth in the first half of 2020 but keeps pace with GDP. Trade growth turns to fall more disproportionately than the activity during the crisis. Global export of goods and services, which had doubled the growth rate of GDP in decades, slowed down during the pandemic relative to economic growth. The stagnation in FDI in industrial manufacturing capacity had a key impact on global trade slowdown (UNCTD, 2020).

The top 500 global multinational companies accounting for global FDI revised project earnings to fall by ~400 bps, with some sectors recording losses. This indicates that reinvestment surplus earning is affected by lower recorded profit, which accounts for 50% of FDI.

**FIGURE 5:**
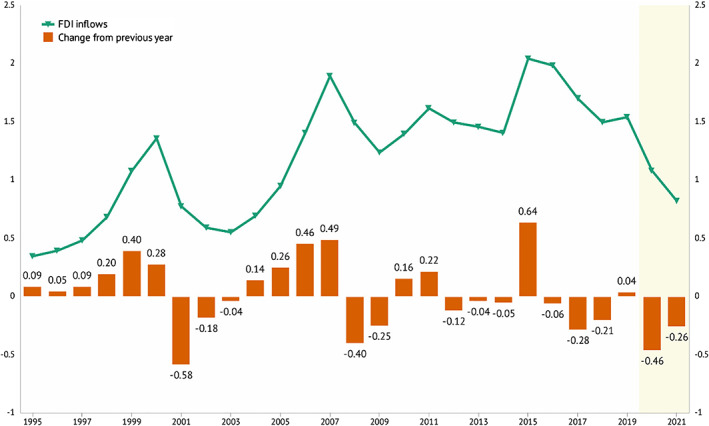
Effect of COVID‐19 pandemic on global FDI inflows. Data source: UNCTD; Knoema (2020)

Figure 5 indicates the change in inflows of global FDI, with 2020 and 2021 recording −42% and −26% changes in FDI inflows, respectively. The expected level of FDI was projected to decline from $2 trillion in 2015 to $1 trillion in 2020 and $900 billion in 2021. The FDI inflow is projected to fall by 2000–3500 bps in developed economies and 2500–4000 bps in Africa—estimated with a major impact from low commodity prices because of the continent's natural resources‐oriented investment. FDI inflows in 2020 fiscal year was estimated to fall by 3000–4500 bps in developing Asia and 5000 bps in Latin America and the Caribbean.

**FIGURE 6:**
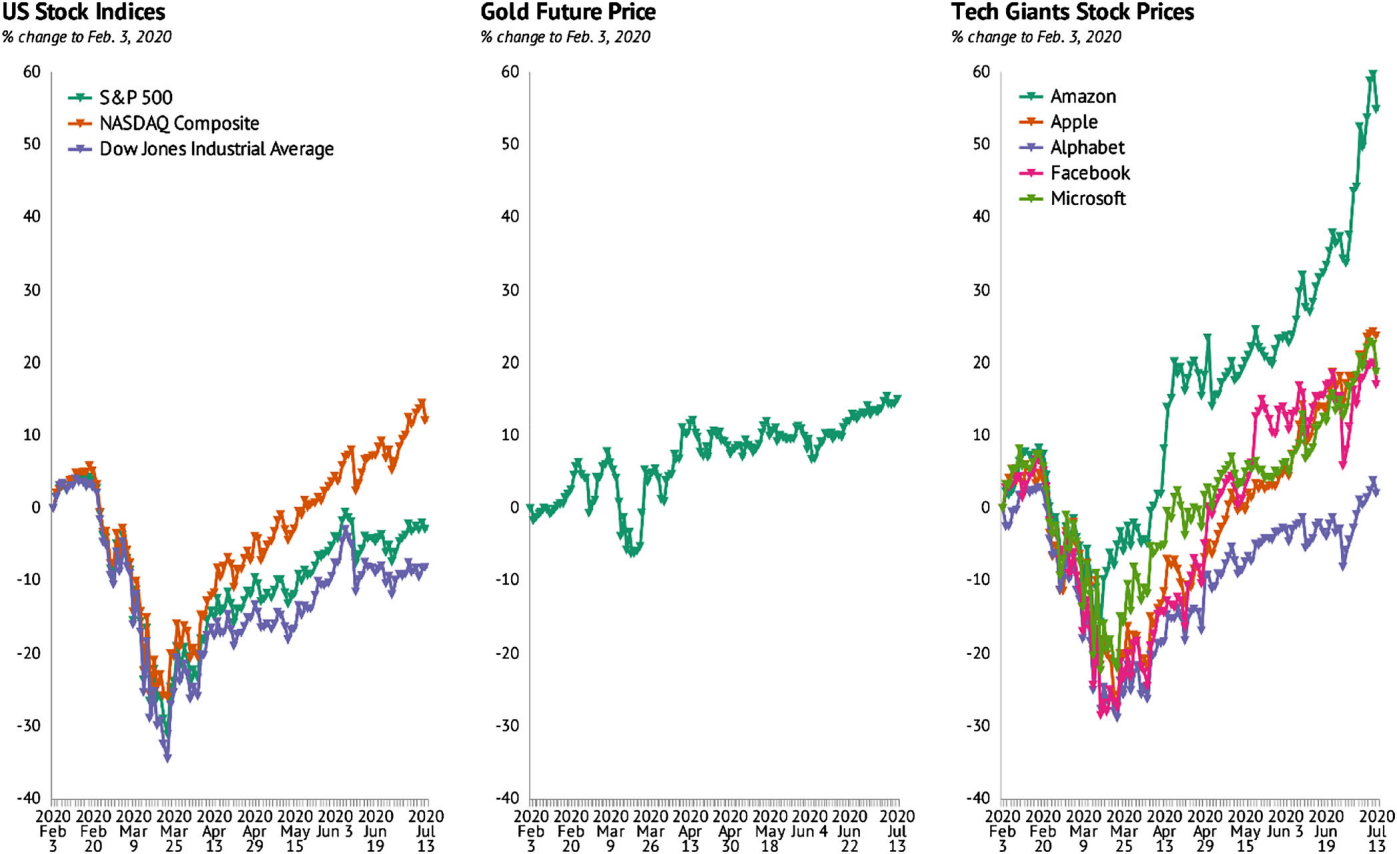
Effect of COVID‐19 on US stock, gold, and tech giants stock. Data source: Investing.com; Knoema (2020)

In contrast, COVID‐19 attributed movement restrictions and social distancing measures have enhanced the global demand for digital goods and services, hence, leading to a hike in stock prices of Tech Giants like Amazon, Apple, Alphabet, Facebook, and Microsoft (Figure 6). Similarly, gold price and the US stock indices such S&P 500, NASDAQ composite, and Dow Jones Industrial average have experienced substantial growth. This implies that while COVID‐19 pandemic is deteriorating in‐person trade of goods and services, a door of opportunity to stimulate the agenda towards digital transformation is on the rise.

In conclusion, despite the unprecedented macroeconomic policy supporting the decline in global FDI, GDP, and trade amidst COVID‐19 pandemic, economic policies will not be sustainable in the long‐term if restrictions and containment measures are not eased. It is observed that many large economies are at risk of gross government debts, which may hamper sustainable economic development. However, in an upside scenario where all pandemic control measures are lifted, a rebound effect of economic development is possible if fiscal and monetary policies, consumer support, and confidence in financial investors are normalized. The unprecedented economic threat presented by COVID‐19 has disrupted the global value chain creation, demand, and supply—that have threatened the survival of businesses. Governments could implement policy support for multinational enterprises (MNEs), especially in developing countries through the provision of tax reliefs in the form of tax wavier, tax credit, and tax deferral. The Mobility of financial support in the form of grants, subsidies, and low‐interest‐credit for the MNEs will assist in compensating for the fall in cash flow. Government and policymakers could introduce flexible regulatory approach to cut down costs, extension of regulatory deadlines for filings, and other support in terms of permit, license, and fee waiver. The government could start strategic planning of economic policies to boost the confidence of investors and guide their transition to the post‐COVID‐19 economic recovery. The robust government policies must be adequate to safeguard the needs of MNEs to ensure FDI inflows that are key to long‐term economic growth and sustainability in developing countries. Further research could explore the impact of COVID‐19 on access and cost of capital in global financial market.

## Data Availability

The data that support the findings of this study are available in Knoema at https://knoema.com/. These data were derived from the following resources available in the public domain: United Nation Conference on Trade and Development. Retrieved from: https://unctad.org/en/pages/PublicationWebflyer.aspx?publicationid=2769; Center for Systems Science and Engineering at John Hopkins University, blog Post. Retrieved from https://buff.ly/2O69IR8.
